# CULTURAL AND SOCIAL FACTORS IN CARE DELIVERY BY AFRICAN AMERICAN CAREGIVERS OF ADULTS WITH DEMENTIA AND COVID-19

**DOI:** 10.1093/geroni/igac059.2986

**Published:** 2022-12-20

**Authors:** Idorenyin Udoh, Elias Mpofu

**Affiliations:** University of North Texas, Denton, Texas, United States; University of North Texas, Denton, Texas, United States

## Abstract

African American /Black caregivers make up 13% of the total number of adult caregivers in the United States and 41% more likely to provide help greater than three activities of daily living than other ethnicities. How they perceive socio-cultural factors to influence their care giver roles is less documented. This study aims to synthesize the evidence on the role of culture and social factors in African American caregiver perceptions delivering care to older adults with dementia and COVID-19. Searches of the Ageline, Medline, PsycInfo, Academic search complete Psychology and Behavioral science collection and Google scholar for empirical study publications on socio-cultural factors for dementia care and COVID-19 among African American caregivers yielded six studies. To be included, studies met the following criteria (a) focus on African American caregivers of older adults with dementia and focus on Covid-19 (b) socio-cultural factors (c) perceptions and practices. (d)published between 2019–2022. Studies indicate compassionate care practices by African American caregivers of persons with dementia and Covid-19. African American care givers of persons with dementia and COVID19 perceive caregiving as a responsibility they owe and not a job. They also perceived to be guided by their racial identity and faith beliefs, integrating family values and culture into caregiving. African American carers of persons living with dementia and COVID-19 have compassion and resilient care self-perceptions in caregiving to people with Dementia and COVID-19. Supporting compassionate care delivery by African American carers requires understanding social and cultural factors driving their commitment to quality care.

